# Construction of Time-Resolved Luminescence Nanoprobe and Its Application in As(III) Detection

**DOI:** 10.3390/nano10030551

**Published:** 2020-03-19

**Authors:** Teng Chen, Haitao Wang, Zhouping Wang, Mingqian Tan

**Affiliations:** 1 School of Food Science and Technology, National Engineering Research Center of Seafood, Collaborative Innovation Center of Seafood Deep Processing, Dalian Polytechnic University, Dalian 116034, China; chenteng_dlpu@163.com; 2 School of Food Science and Technology, Jiangnan University, Wuxi 214122, China

**Keywords:** As(III), time-resolved luminescence, aptamer, nanorods

## Abstract

As(III) is a toxic heavy metal which causes serious health problems. Therefore, the development of highly sensitive sensors for As(III) detection is of great significance. Herein, a turn-on luminescence resonance energy transfer (LRET) method based on luminous nanorods was designed for As(III) detection. Biotin-labelled As(III) aptamers were tagged to avidin functionalized luminous nanorods as energy donors, while graphene oxide (GO) acted as the energy acceptor. The adsorption of single-stranded DNA on graphene oxide resulted in the efficient quenching of the luminescence of the nanorods through the LRET process. In the presence of As(III), aptamers bonded to As(III) preferentially and resulted in the formation of aptamer-As(III) complexes. The aptamer-As(III) complexes were rubbed off from the GO surface due to their conformational change, which led to the recovery of the luminescence of the nanorods. A good linear relationship between the luminescence intensity and concentration of As(III) was obtained in the range from 1 to 50 ng·mL^−1^, with a detection limit of 0.5 ng·mL^−1^. Furthermore, the developed sensors showed good specificity towards As(III) and proved capable of detecting As(III) in the environment and food samples. The proposed time-resolved sensors provide a promising sensing strategy for the rapid and sensitive detection of As(III).

## 1. Introduction

As(III) is a toxic heavy metal, which is about 60 times more toxic than other forms of arsenic [1]. Because of the slow excretion of As(III) after entering the human body, long-term accumulation in the body can cause arsenic poisoning. The repeated occurrence of arsenic pollution incidents has seriously threatened people’s life and physical health; caused various diseases such as cancer [2] and cardiovascular and nervous system diseases, and has become a problem that cannot be ignored in food safety. Inorganic arsenic is mostly found in environmental water: the World Health Organization (WHO) has set the upper limit for the content of As(III) in environmental water to 10 ppb [3]. Although organic arsenic is the main form of arsenic in aquatic products [4], studies have shown that the content of inorganic arsenic in shellfish living in arsenic-contaminated waters has increased significantly, and the toxicological problems caused by inorganic arsenic still cannot be ignored [5,6].

An aptamer is a single-stranded oligonucleotide that can specifically bind to a target molecule with high affinity [7,8] and is usually artificially synthesized in vitro using a systematic evolution of ligands by exponential enrichment (SELEX) [9]. Aptamers are suitable for a wide range of targets due to the different spatial structures of their respective bases [10]. Due to the ability of aptamers to bind to specific targets, many scholars have have established a variety of detection methods for hazardous substances in different foods, including biotoxins [11], foodborne pathogens [12], pesticide and veterinary drug residues [13], heavy metals [14,15] and illegal additives [16]. 

‘Persistent luminescent nanoparticles’ (PLNPs) refers to a series of nanomaterials that can continuously emit luminescence after being excited [17]. Their luminescence decay time ranges from a few microseconds to several hours [18]. Since the autofluorescence lifetime of the complex system is one nanosecond or less, the decay of the background noise will soon be completed after the excitation light is turned off [18]. In contrast, the PLNPs can continuously maintain a higher intensity luminescence, thereby avoiding the interference of autofluorescence and improving the signal-to-noise ratio [17,19]. Tan’s group has reported the hydrothermal synthesis of Zn_2_GeO_4_:Mn (ZGO:Mn) nanorods of different sizes by adjusting the pH of the system [20]. Unlike traditional solid-phase calcination, ZGO:Mn nanorods prepared using hydrothermal synthesis disperse better in aqueous solutions, which is more suitable for the fabrication of As(III) probes [21]. Recently, with the development of the theory, more and more optical sensors for As(III) detection based on various principles have been designed [22], including fluorescent [23,24,25,26], colorimetric [27,28], resonance Rayleigh scattering (RRS)-based [29], chemiluminescence-based [30,31] and electrochemical [31] methods. However, there are few reports of the development of As(III) time-resolved luminescence probes based on aptamer-modified PLNPs. 

**Scheme 1:**
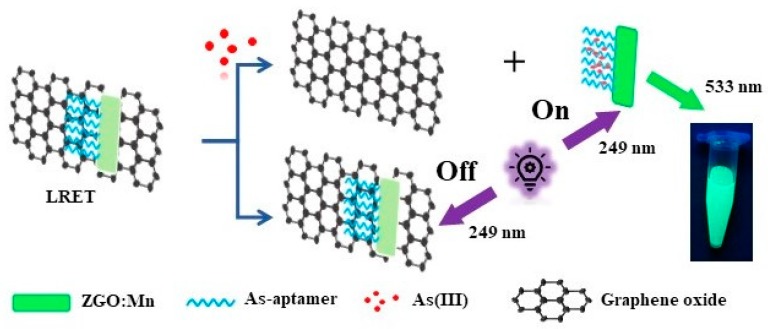
The detection principle of As(III) by the time-resolved fluorescent probe via a luminescence resonance energy transfer (LRET).

In this study, we constructed a time-resolved luminescence probe for As(III) using green florescent PLNPs as signal emitters, based on the specific binding mechanism of the aptamer for As(III). As shown in Scheme 1, the aptamer-modified long-lived PLNPs were attached onto graphene oxide (GO) through π-π adsorption [32,33,34,35]. Their luminescence was quenched via a turn-on luminescence resonance energy transfer (LRET) mechanism between the PLNPs and GO. When the target As(III) was added to the system, the aptamers bond to the As(III) to form aptamer-As(III) complexes. Since the affinity of the aptamer and the As(III) is much higher than that of ssDNA and GO, the PLNPs detached from the GO surface due to the conformational change, which led to the luminescence recovery of the nanoparticles. By exploring the relationship between the degree of luminescence recovery and the concentration of the target substance, the quantitative analysis of As(III) was realized by measuring the luminescence signal. 

## 2. Results

### 2.1. Characterization of the ZGO: Mn

**Figure 1:**
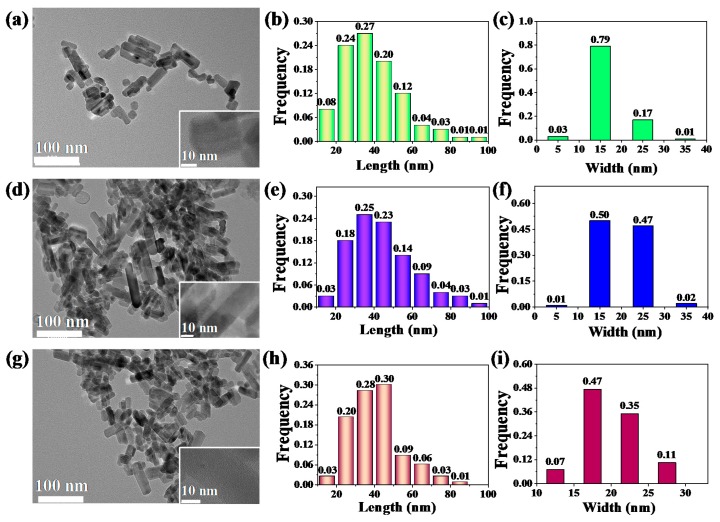
TEM of the nanorods with different Mn^2+^ addition and statistical results of their length and width (0.1% Mn^2+^: (**a**–**c**); 0.25% Mn^2+^: (**d**–**f**); 0.5% Mn^2+^: (**g**–**i**)).

The morphology and crystal structure of Zn_2_GeO_4_:Mn (ZGO:Mn) were characterized by TEM and XRD techniques. As shown in Figure 1, the sizes of the prepared Zn_2_GeO_4_:Mn fluorescent materials were all at the nanometer level, and a single particle exhibited a regular rod-like shape. It was found that the length and width of ZGO:Mn increased with the increase of doped Mn^2+^ based on the statistic results of 100 nanorods. The proportion of nanorods with a length of 45 nm increased from 0.20 to 0.30, while the ratio of nanorods with a width of 15 nm decreased from 0.79 to 0.47. On the other hand, the number of nanorods with a width greater than 15 nm increased significantly. In short, the aspect ratio of the prepared ZGO: Mn was reduced as the Mn^2+^ increased from 0.1% to 0.5%. The nanorods with 0.5% Mn^2+^ doping are more uniform and are easily dispersed in aqueous solution (Figure S1).

**Figure 2:**
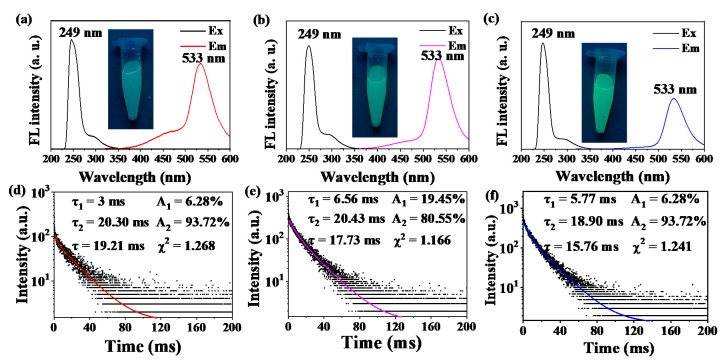
The luminescence spectra and the luminescence lifetime of the ZGO: Mn with different Mn^2+^ (0.1% Mn^2+^: (**a**,**d**); 0.25% Mn^2+^: (**b**,**e**); 0.5% Mn^2+^: (**c**,**f**)). The illustrations in (**a**–**c**) are photos of the PLNPs under 254nm excitation.

From the luminescence spectra of ZGO:Mn with different Mn^2+^ (Figure 2a–c and Figure S2), the nanorods exhibit maximum excitation at 249 nm and maximum emission wavelength at 533 nm regardless of the different concentrations of Mn^2+^. The green emission at 533 nm is attributed to the escaped electrons moved by the excited energy level of Mn^2+^, resulting in the recombination of the electrons with holes that are generated by the nanorods ZGO:Mn under UV excitation [20]. The insets show photographs of the nanorods which emit strong green luminescence. The Stokes shift of three types of nanorods is 284 nm, which is beneficial for overcoming the shortcoming of excitation wavelength interference in time-resolved luminescence detection [32,33]. Moreover, the peak width at half height for all the nanorods is around 55 nm, suggesting that the emission peak of the ZGO:Mn is sharp. The luminescence lifetimes of the ZGO: Mn with 0.10%, 0.25% and 0.50% Mn^2+^ are 19.21, 17.73 and 15.76 ms (χ^2^ < 1.3), respectively. The lifetime of a millisecond is sufficient for the application of time-resolved luminescence assay [36]. In this study, ZGO: Mn doping with 0.5% Mn^2+^ was selected for the following experiment. 

**Figure 3:**
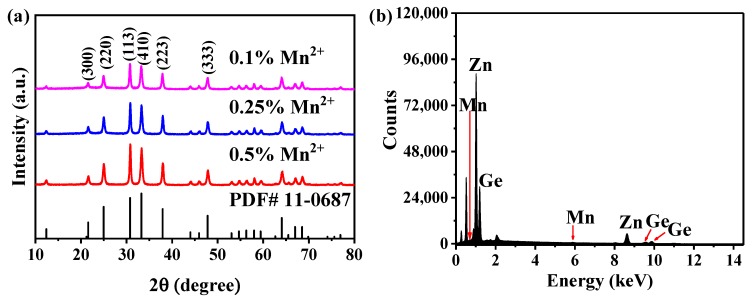
(**a**) XRD spectra of Mn^2+^-doped ZGO: Mn with 0.1%, 0.25% and 0.5% Mn. (**b**) EDS spectrum of Zn_2_GeO_4_ containing 0.5% Mn (ZGO:0.5% Mn).

Figure 3a shows the XRD spectra of different Mn^2+^ -doped ZGO: Mn. All the diffraction peaks of the sample match the data of the Zn_2_GeO_4_ standard card (PDF # 11-0687), and no impurity peak is observed, indicating that the incorporation of manganese ions at low concentrations did not significantly change the crystal structure of Zn_2_GeO_4_. All the prepared nanorod materials are single phase. However, in the enlarged view of 2 theta between 30°–40°, a slight shift of peak position can be observed (Figure S3) due to the unit cell size change with different incorporated-Mn^2+^. In addition, the intensity of the diffraction peak of the sample gradually decreased, indicating a less layered structure after the hydrothermal reaction. Energy-dispersive X-ray spectroscopy (EDS) analysis of Zn_2_GeO_4_:Mn (ZGO: Mn) showed that the prepared nanomaterials contained matrix elements from zinc, germanium and oxygen, and doping element manganese ions, which further proved the successful incorporation of manganese into ZGO: Mn nanorods (Figure 3b and Figure S4).

### 2.2. Fabrication of the As(III) Probe

**Figure 4:**
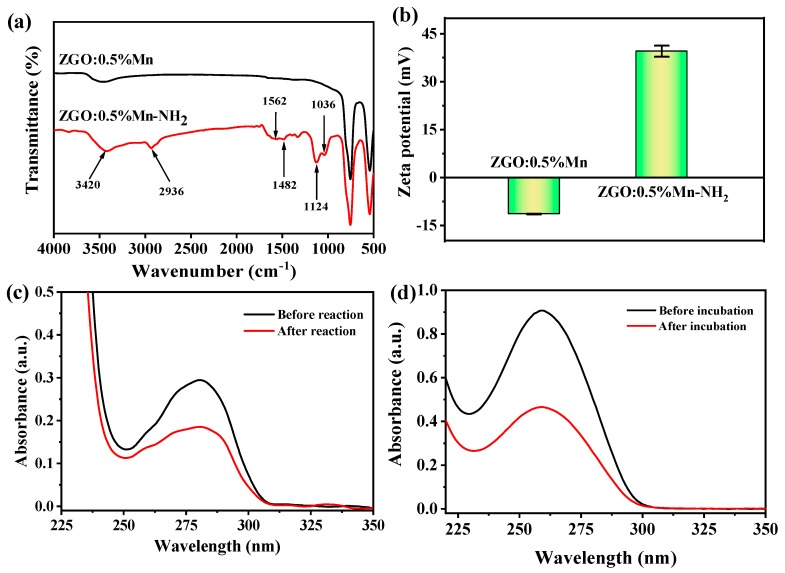
(**a**) FT-IR spectra of the ZGO:0.5%Mn before and after amination. (**b**) The change of Zeta potential before and after the amination treatment. (**c**) UV-vis spectra of the avidin solution before and after binding with ZGO:0.5%Mn-NH_2_. (**d**) UV-vis spectra of the biotin-labelled aptamer supernatant before and after incubation with ZGO:0.5%Mn-avidin.

Figure 4a shows the Fourier Transform Infrared (FT-IR) spectra of ZGO:0.5%Mn before and after modification with (3-aminopropyl) triethoxysilane (APTES). It is noteworthy that a series of new characteristic absorption peaks of APTES appeared in the FT-IR spectrum. Stretching and bending characteristic peaks at 3420 cm^−1^ and 1562 cm^−1^ are derived from the amino group. The vibration peak at 2936 cm^−1^ comes from the stretching modes of C–H, and the absorption in the range of 1490–1530 cm^−1^ comes from the bending vibration of C–H. The stretching absorption peak of C–N occurred at 1124 cm^−1^, and stretching of Si–O at 1036 cm^−1^ was also observed. The FT-IR results indicate that the characteristic absorption peaks were all from the APTES, and the APTES molecules were successfully conjugated onto the surface of the nanorods ZGO:0.5%Mn. In addition, the zeta potential of the ZGO:0.5%Mn increased from −11.42 mV to 39.55 mV after the amination treatment, which further verified the successful conjugation of the amino group on the ZGO:0.5%Mn surface.

To further conjugate the avidin onto ZGO:0.5%Mn-NH_2_, glutaraldehyde was used to prepare avidin-coated fluorescent nanomaterial ZGO:0.5%Mn-Avidin, since amino groups could react with aldehyde groups and form a Schiff base. The UV-vis spectra in Figure 4c show that the absorption peak of avidin at 280 nm did not shift before and after reaction with ZGO:0.5%Mn-NH_2_. The absorption peak of ZGO:0.5%Mn-NH_2_ was measured, however no useful information was obtained due to the strong interference of the absorption for ZGO:0.5%Mn-NH_2_. We noted that the intensity of the characteristic absorption peak at 280 nm before interaction with ZGO:0.5%Mn-NH_2_ was 0.294, which decreased to 0.185 for the supernatant obtained by centrifugation. This indicates that a large amount of avidin is successfully attached to the surface of ZGO:0.5%Mn-NH_2_. Similarly, the intensity of the UV-vis absorption peak at 260 nm for the biotin-labelled aptamer supernatant was 0.906, and it decreased to 0.465 after the interaction between biotin and avidin (Figure 4d). The significant decrease of the absorption peak intensity indicates that the biotinylated aptamer was successfully attached to the surface of ZGO:0.5%Mn-avidin.

### 2.3. Optimization of Experimental Conditions

**Figure 5:**
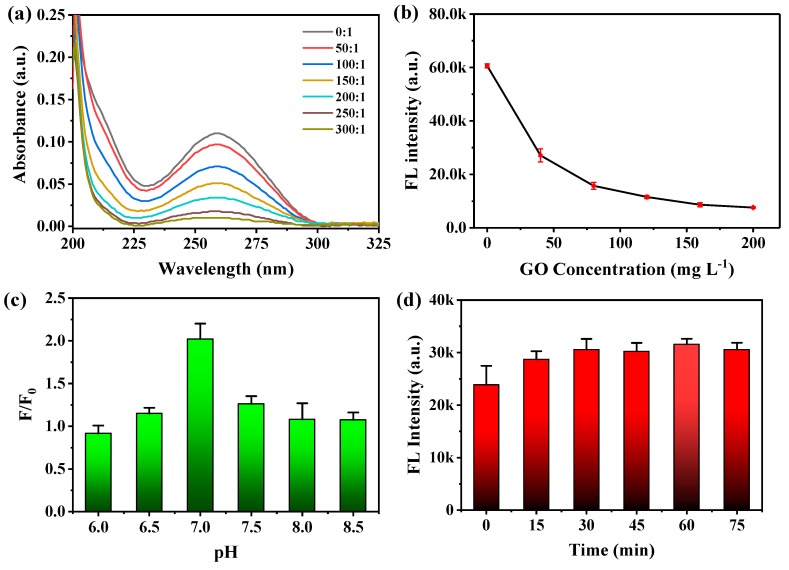
Optimization of experimental conditions. (**a**) UV-vis spectra of the aptamers supernatant after 30 min incubation at different ZGO:0.5%Mn/aptamer mass ratios of 0:1, 50:1, 100:1, 150:1, 200:1, 250:1, and 300:1 (wt%). (**b**) Effect of GO concentration on the luminescence intensity of ZGO:0.5%Mn-aptamer. (**c**) The *F/F_0_* value change of the aptamer-loaded ZGO:0.5%Mn at different pH (F_0_ and F, represents the luminescence intensity of the nanoprobe in the absence and presence of adenosine, respectively.) (**d**) Effect of As(III) incubation time on luminescence intensity of ZGO:0.5%Mn-aptamer.

Since the binding capacity of avidin and biotin is limited, the excessive use of biotinylated aptamer results in a decline in sensitivity. The amount of aptamer was optimized firstly to achieve sensitive detection. The ZGO:0.5%Mn-avidin and aptamers were incubated at different mass ratios: 0:1, 50:1, 100:1, 150:1, 200:1, 250:1, and 300:1. After the reaction, the UV-vis absorption of the supernatant of each condition was detected. As shown in Figure 5a, the characteristic absorption peak of the aptamer gradually decreased at 260 nm as the amount of ZGO:0.5%Mn-avidin increased, indicating that there are fewer free aptamers in the solution, and more aptamers are bonded onto the ZGO:0.5%Mn surface. When the mass ratio reached 300:1, aptamer incubation was close to saturation, so a mass ratio of 300:1 was used for aptamer addition. The pH effect on the signal-to-noise ratio of the nanorods probe was then investigated. 

As shown in Figure 5b, with the increase of the concentration of the GO quencher, the luminescence intensity of the nanoprobe continuously decreases. The most significant attenuation of luminescence intensity was observed when the GO concentration was 40 mg·L^−1^. Moreover, when the pH value increased from 6.0 to 8.5, the *F/F_0_* value first increased and then decreased, and the maximum value was obtained at pH 7.0 (Figure 5c). In addition, to reduce the time taken for detection, the target As(III) incubation time was also optimized. When the incubation time exceeds 30 minutes, the luminescence intensity increases (Figure 5d). Therefore, 40 mg·L^−1^ GO, pH 7.0 of phosphate-buffered saline (PBS) buffer and an incubation time of 30 min were used as the optimal reaction conditions in the following study.

### 2.4. Sensitivity and Selectivity of As(III) Detection 

**Figure 6:**
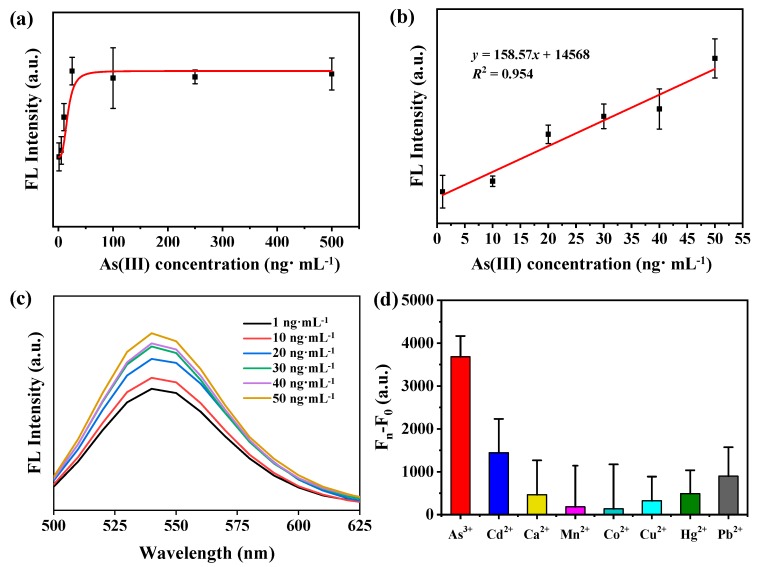
(**a**) The luminescence recovery curve of the probe in the As(III) concentration range of 1–500 ng·mL^−1^. The luminescence recovery curve (**b**) and luminescence spectra (**c**) of the nanoprobe in the presence of As(III) range from 1 to 50 ng·mL^−1^; (**d**) Response of nanoprobe to different interfering ions, the concentration of As(III) was 10 ng·mL^−1^, *F_0_* represents the luminescence intensity before the addition of ion while *F_n_* was the luminescence after incubated with ions.

The luminescence intensity increases significantly with the increase of the target As(III) concentration (Figure 6a). The luminescence intensity showed a linear relationship with the level of As(III) in the range from 1 ng·mL^−1^ to 50 ng·mL^−1^ (Figure 6b–c). After the concentration of As(III) exceeds 100 ng·mL^−1^, the luminescence reaches its plateau and remains unchanged with the increase of As(III). The linear regression equation was *y*= 158.57*x* + 14568 with a correlation coefficient (*R*^2^) of 0.954. The limit of detection (LOD) was calculated (LOD = 3 *σ/S*, *σ* is the standard deviation of the blank sample, and *S* was the slope of the fitted curve) to be 0.5 ng·mL^−1^ (6.5 nM), which was lower than the upper limit of 10 ng·mL^−1^ specified by the WHO. Furthermore, the analytical performance of this method has been compared with those reported in previous studies (Table 1). The LOD in this work is relatively small compared with that of the listed methods. To verify the specificity of this nanoprobe, the luminescence response of the nanoprobe to a series of common metal ions was investigated. The results suggest that the developed nanoprobe was almost unaffected by metal ions of Cd^3+^, Ca^2+^, Mn^2+^, Co^2+^, Cu^2+^, Hg^2+^ and Pb^2+^, since the luminescence recovery could be ignored.

### 2.5. Testing of Actual Samples 

The As(III) content of lake water, packaged drinking water and scallop meat was determined to explore the potential applications of the time-resolved fluorescent nanoprobe for As(III) detection in actual samples. As shown in Table 2, the recoveries of the As(III) range from 119.00% to 133.24%, and the corresponding relative standard deviations (RSD) range from 15.0% to 21.5%. The relatively lager RSD indicates that this method still needs to be optimized before its application for As(III) detection in actual samples. Overall, these results show that the nanoprobe developed in this study may have potential for the quantitative detection of As(III) in real samples. 

## 3. Experimental

### 3.1. Materials and Instruments 

Zinc nitrate, manganese nitrate, germanium oxide, and (3-Aminopropyl)triethoxysilane (APTES) were purchased from Aladdin Biochemical Technology Co., Ltd (Shanghai, China). Avidin and aptamer with a sequence of 5’-TTACAGAACAACCAACGTCGCTCCGGGTACTTCTTCATCG-Biotin-3’ [29] were purchased from Shanghai Sangon Biological Engineering Technology (Shanghai, China). Graphene oxide was purchased from Nanjing Xianfeng Nanomaterial Technology Co., Ltd (Nanjing, China). The standard solution (1000 μg·mL^−1^) was purchased from Beijing Putian Tongchuang Biological Technology Co., Ltd. (Beijing, China), and the remaining analytical grade chemical reagents were purchased from Sinopharm Chemical Reagent Co., Ltd (Shanghai, China). The ultrapure water (18.2 mΩ) used throughout the experiment was provided by a Millipore water purification system (Millipore, Bedford, MA, USA).

Transmission electron microscopy (TEM) images of the PLNPs were characterized by a JEM-2100 transmission electron microscope (JEOL., Tokyo, Japan). Luminescence spectra and luminescence lifetime were measured with a FLS980 fluorescence spectrometer (Edinburgh Instruments Co., Livingston, UK). X-ray diffraction (XRD) spectrum information was obtained by XRD-6100 X-ray diffractometer (Shimadzu, Japan). X-ray energy spectra (EDS) was obtained by an X-Max50 spectrometer (Oxford Instruments Co., Oxford, UK). Ultraviolet-visible (UV-vis) absorption spectra were recorded by a Lambda 35 UV-Vis spectrometer (PerkinElmer, San Jose, CA, USA). The change in Zeta potential was verified by a Zeta sizer Nano ZS03031301 (Malvern, Worcestershire, UK). A Spectra Max M5/M5e microplate reader (Molecular Devices, San Jose, CA, USA) was used for the detection of As(III).

### 3.2. Preparation of the Zn_2_GeO_4_: Mn (ZGO: Mn)

In this study, ZGO: Mn with different Mn^2+^ contents (0.1%, 0.25%, 0.5%) were prepared. Taking ZGO:0.5%Mn as an example, 2 mmol Zn(NO_3_)_2_ and 0.005 mmol Mn(NO_3_)_2_ were dispersed in 10 mL of ultrapure water using sonication, 300 μl of concentrated HNO_3_ was added to this solution under vigorous stirring. After 1 mmol of Na_2_GeO_3_ was added dropwise to the above solution, the pH of the mixture was immediately adjusted to 9.5 using ammonium hydroxide (28%, wt%). After stirring at room temperature for 1 h, the mixed solution was transferred to a Teflon-lined reactor and reacted at 220 °C for 4 h. The reaction solution was cooled to room temperature, centrifuged and washed with ultrapure water. The ZGO: Mn nanomaterials were obtained by vacuum freeze-drying.

### 3.3. Surface Modification of the ZGO: Mn

Firstly, the as-prepared ZGO: Mn was modified using APTES, and the resulting ZGO: Mn-NH_2_ was dispersed in ultrapure water [20]. Secondly, avidin-coated ZGO: Mn (ZGO: Mn-Avidin) was synthesized through the addition of glutaraldehyde [37]. Finally, the aptamer was immobilized on ZGO:0.5%Mn-avidin using biotin-avidin interaction. The ZGO: Mn-aptamer was re-dissolved in PBS buffer and stored at 4 °C for later use. 

### 3.4. Detection of As(III) Standard Solution

Here, graphene oxide (GO) was used as a black hole quencher. 100 μL ZGO:0.5%Mn-aptamer (0.5 mg·mL^−1^) and 100 μL graphene oxide (120 mg·L^−1^) solution were thoroughly mixed, and the above solution was reacted at 25 °C for 30 minutes. After incubation, 100 μL of As(III) standard solutions of different concentrations were directly added to the system, and 100 μL of the PBS buffer was added to make up the volume to 400 μL. The above solution was further incubated at 25 °C for 30 min after vigorous shaking. After reaction, the measurement was performed using the time-resolved mode of the Spectra Max M5/M5e microplate reader. Under the excitation of a wavelength of 250 nm, 500 μs signal collection after 100 μs delay, the time-resolved fluorescence intensity of the solution at 540 nm was detected.

### 3.5. Preparation of Actual Samples

The lake water was collected in Jiangnan University (Wuxi, China), and filtered through a 0.22 μm membrane to remove particulate impurities. Packaged drinking water and scallop meat were purchased from a local supermarket (Dalian, China). The scallop meat was washed with deionized water, and the excess water was filtered off before homogenizing. 1.0 g of the homogenized sample was dispersed in 20 mL of 0.15 M nitric acid, mixed by shaking and left overnight. The mixture was hot-extracted at 90 °C for 2.5 h, and the extract was centrifuged after cooling to room temperature. Excess fat was removed from the supernatant with n-hexane and further filtered using a 0.22 μm membrane. The resulting filtrate was adjusted to a neutral pH and diluted with a pH 7.0 PBS buffer (1:5, *v*:*v*). For the preparation of spiked samples, the prepared 100 μg mL^−1^ As(III) stock solution was added to these three samples before pretreatment.

## 4. Conclusions

In the present study, the aptamer probe for As(III) detection was constructed successfully. The time-resolved nature of the luminous nanoparticles eliminates the interference caused by autofluorescence. The aptamer was used as the recognition element in these nanoprobes, and exhibited acceptable sensitivity and selectivity. The detection limit for As(III) was 0.5 ng·mL^−1^ (6.5 nM) under the optimal experimental conditions. Moreover, the developed nanoprobes were used to detect As(III) in real samples. By replacing different aptamers, this time-resolved detection method is expected to be extended to the detection of other target substances and play a greater role in the food safety inspection field. 

## Figures and Tables

**Table 1:** Related reports on nanomaterial-based sensors for the detection of As(III).

Signal	Nanomaterial	LOD (ng·mL^−1^)	Water Sample	Food Sample	Reference
Fluorescence	Fe_3_O_4_ NPs	3.75	Yes	No	[23]
Fluorescence	MSNs	0.90	Yes	No	[24]
Fluorescence	ZnO QDs	27	Yes	No	[25]
Fluorescence	MoS_2_	1.35	Yes	No	[26]
Colorimetry	Ag NPs	5.98	Yes	No	[27]
Colorimetry	Au NPs	1.27	Yes	No	[28]
RRS	Ag NPs	1.9	Yes	No	[29]
Fluorescence	ZGO: Mn	0.5	Yes	Yes	This work

**Table 2:** Determination of As(III) in water samples and food sample.

Sample	Added	Found	RSD	Sample
Lake water	50 ng·mL^−1^	63.85 ng·mL^−1^	15.7	127.70
Drinking water	50 ng·mL^−1^	66.62 ng·mL^−1^	15.0	133.24
Scallop meat	1 mg·kg^−1^	1.19 mg·kg^−1^	21.5	119.00

## Supplementary Materials

The following are available online at https://www.mdpi.com/2079-4991/10/3/551/s1, Figure S1: Dispersion of Zn_2_GeO_4_:0.50%Mn (ZGO:0.5%Mn) in aqueous solution; Figure S2: Fluorescence (FL) spectra of Zn_2_GeO_4_:0.10%Mn, Zn_2_GeO_4_:0.25%Mn and Zn_2_GeO_4_:0.50%Mn at same concentration which were excited at 250 nm; Figure S3: Partially enlarged XRD spectra of Zn_2_GeO_4_:0.10%Mn, Zn_2_GeO_4_:0.25%Mn and Zn_2_GeO_4_:0.50%Mn with 2θ in the range of 30°–40°; Figure S4: EDS analysis of (a) Zn_2_GeO_4_:0.10%Mn, (b) Zn_2_GeO_4_:0.25%Mn.
