# Supplementary material for: Construction of Time-Resolved Luminescence Nanoprobe and Its Application in As(III) Detection
Source: Nanomaterials (Basel). 2020 Mar 19;10(3):551. doi: 10.3390/nano10030551 (PMC7153583; doi:10.3390/nano10030551)
Supplement: Supplementary file 1 [file nanomaterials-10-00551-s001.pdf]

nanomaterials

Supplementary Materials:

Construction of Time-Resolved Luminescence Nanoprobe and Its Application in As(III) Detection

Teng Chen [1,2], Haitao Wang [1,*], Zhouping Wang [2,*] and Mingqian Tan [1,*]

1 School of Food Science and Technology, National Engineering Research Center of Seafood, Collaborative Innovation Center of Seafood Deep Processing, Dalian Polytechnic University, Dalian 116034, China; chenteng_dlpu@163.com (T.C.)
2 School of Food Science and Technology, Jiangnan University, Wuxi 214122, China
* Correspondence: wanght@dlpu.edu.cn (H.W.); wangzp@jiangnan.edu.cn (Z.W.); mqtan@dlpu.edu.cn (M.T); Tel.: +86-510-85917023 (Z.W.), +86-411-86318657 (H.W. & M.T.)

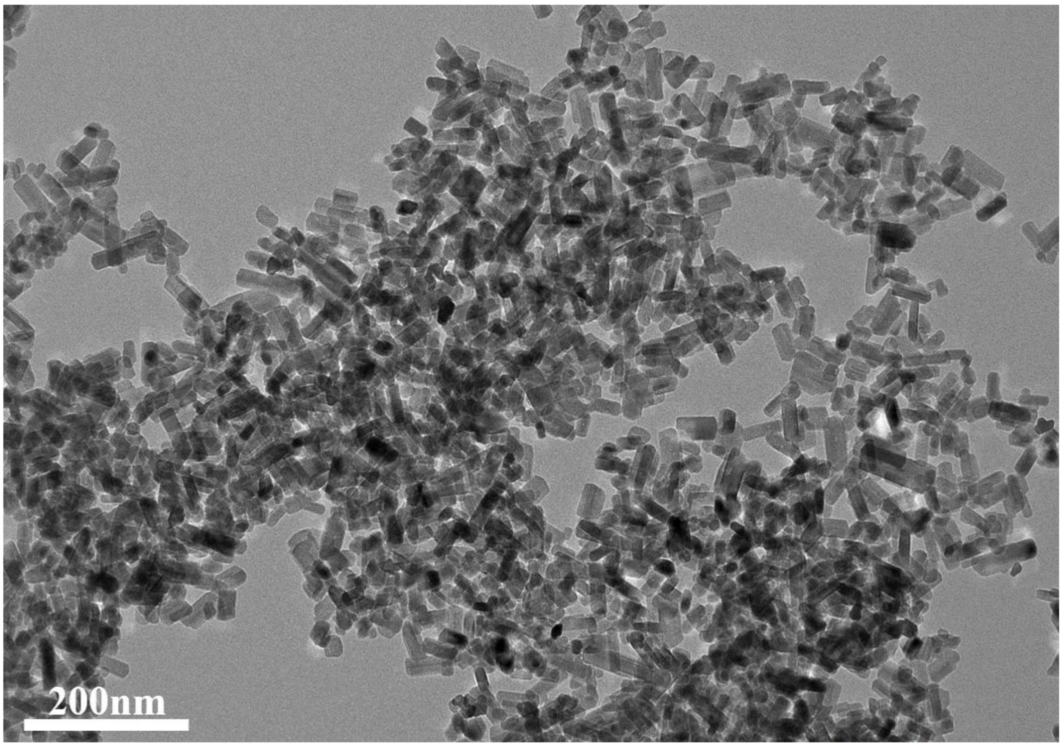

Figure S1. Dispersion of ZGO:0.5% Mn in aqueous solution.

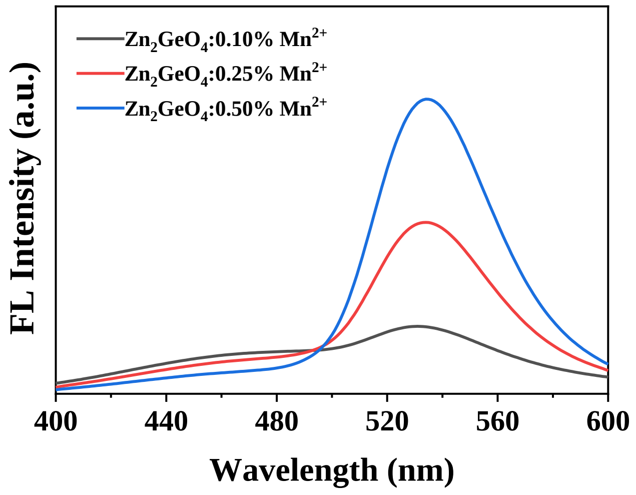

Figure S2. Fluorescence (FL) spectra of Zn_2GeO_4:0.10%Mn, Zn_2GeO_4:0.25%Mn and Zn_2GeO_4:0.50%Mn at same concentration which were excited at 250 nm.

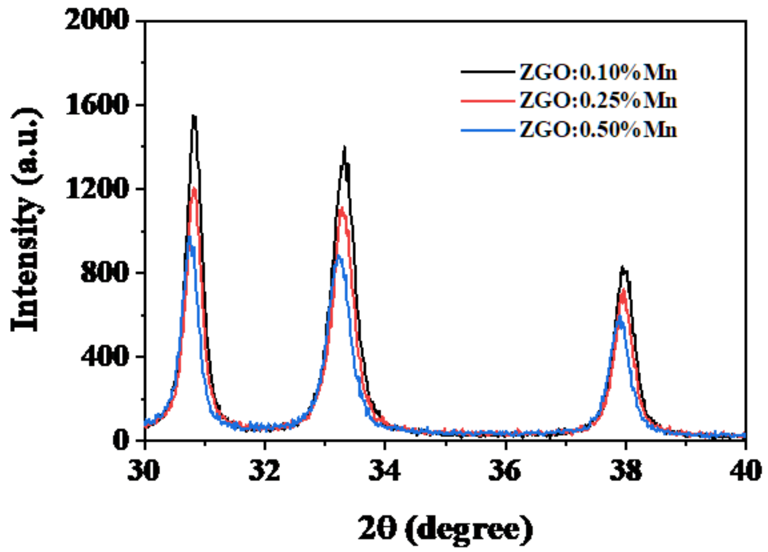

Figure S3. Partially enlarged XRD spectra of Zn_2GeO_4:0.10%Mn, Zn_2GeO_4:0.25%Mn and Zn_2GeO_4:0.50%Mn with 2θ in the range of 30°–40°.

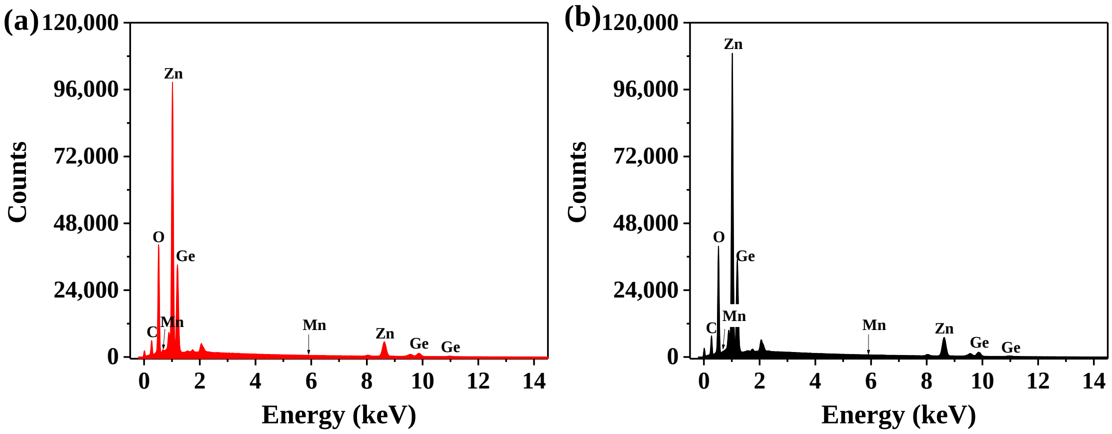

Figure S4. EDS analysis of (a) Zn_2GeO_4:0.10%Mn, (b) Zn_2GeO_4:0.25%Mn.
